# Knee Desmoplastic Melanoma, an Uncommon Tumor, Disguised as a Malignant Peripheral Nerve Sheath Tumor: A Case Report and Review of the Literature

**DOI:** 10.7759/cureus.76524

**Published:** 2024-12-28

**Authors:** Hanan Bailal, Fadoua Jebrouni, Soufia El Ouardani, Ouissam Al Jarroudi, Sami Aziz Brahmi, Said Afqir

**Affiliations:** 1 Medical Oncology, Mohammed VI University Hospital, Oujda, MAR; 2 Medical Oncology, Faculty of Medicine and Pharmacy of Oujda, Mohamed First University, Oujda, MAR

**Keywords:** desmoplastic, diagnosis challenge, immunochemistry, melanoma, surgery

## Abstract

Desmoplastic melanoma is a rare and distinct subtype of cutaneous melanoma, it presents diagnostic challenges due to the lack of specific clinical features and overlapping histopathological characteristics with other malignancies, which necessitate careful clinicopathological correlation and advanced immunohistochemical profiling. While surgical excision remains the cornerstone of treatment, advances in precision medicine, particularly immune checkpoint inhibitors, have shown promise in improving outcomes for unresectable and metastatic desmoplastic melanoma. We present a case study involving a 52-year-old woman misdiagnosed with a malignant peripheral nerve sheath tumor and later identified as desmoplastic melanoma through re-evaluation of histopathological and immunohistochemical findings. The patient underwent surgical resection followed by adjuvant radiotherapy and showed favorable locoregional development.

## Introduction

Cutaneous melanoma is a malignant tumor that arises from the excessive activation or genetic alteration of epidermal melanocytes, leading to abnormal cell growth [1,2]. This type of cancer belongs to a broad category of malignancies, characterized by various features such as epidemiology, morphology, genetic profile, and biological behavior [3]. To date, histological examination remains the gold standard for melanoma diagnosis, with the use of immunohistochemistry being unavoidable [4].

Desmoplastic melanoma (DM) is a rare subtype of melanoma representing less than 4% of all cutaneous melanomas [5]. It was initially reported in the literature by Conley et al. in 1971 [6]. DM often represents a major diagnostic challenge to physicians due to the lack of specific characteristics to help in clinical diagnosis, and to pathologists who should be suspicious about its pathological features and its immunohistochemical profile that might be similar to conventional types of melanoma [7], and other malignancies [8,9]. Typically, the clinical presentation of DM includes non-pigmented lesions showing locally aggressive growth with a predisposition for local recurrence, less frequent nodal metastasis, and more frequent visceral metastases [10].

In 1979, Reed and Leonard discovered neurotropism as a common characteristic of DM [11]. This refers to the development of a tumor in the perineural or intraneural space. Management of DM contains several therapeutic options, with surgical resection as the main one. Systemic and radiation therapy are also to be mentioned in this field. However, in recent years, as precision medicine has advanced, we are increasingly witnessing a wide range of indications for targeted therapies and immunotherapy, including this tumor [12].

This case study features a patient who had a right knee mass that was first identified as a malignant peripheral nerve sheath tumor (MNPST). However, a further immunohistochemical examination revealed a change in the diagnosis, which was unexpected, to a DM.

## Case presentation

**Figure 1:**
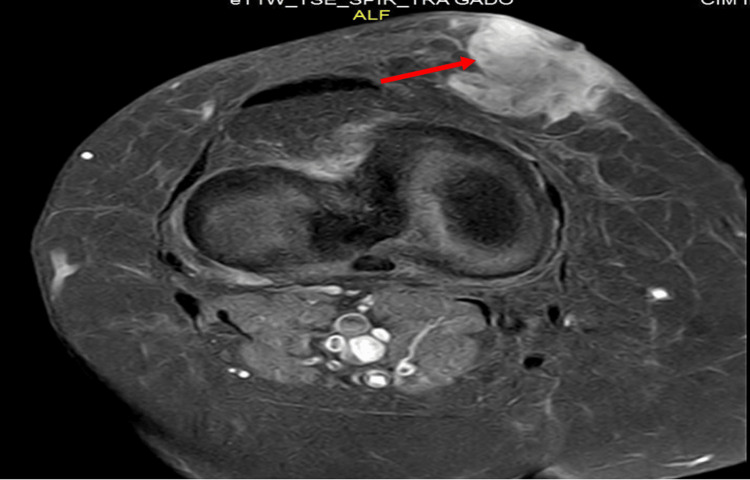
Knee MRI showing a heterogeneously enhancing deep and superficial tissue mass (Red arrow)

A 52-year-old woman with a medical history of burns on both lower limbs 26 years ago and a thyroidectomy in 2013, who was taking levothyroxine, was referred to the oncology department to supplement the management of a resectable MNPST. A painless, gradually growing mass in the right knee, without any accompanying inflammatory symptoms, was the initial lesion for more than five years. It was first removed a year and a half ago without an anatomopathological examination of the removed portion. The evolution was marked by the recurrence in the same location of a painless white nodule that was gradually growing in bulk and had the same clinical characteristics as the prior lesion. An MRI of the knee revealed a heterogeneously enhancing deep and superficial tissue mass measuring 29x31x34 mm in the medial soft tissues of the right knee opposite the kneecap, without a hemorrhagic component (Figure 1). The extension work-up showed no evident signs of distant metastases.

**Figure 2:**
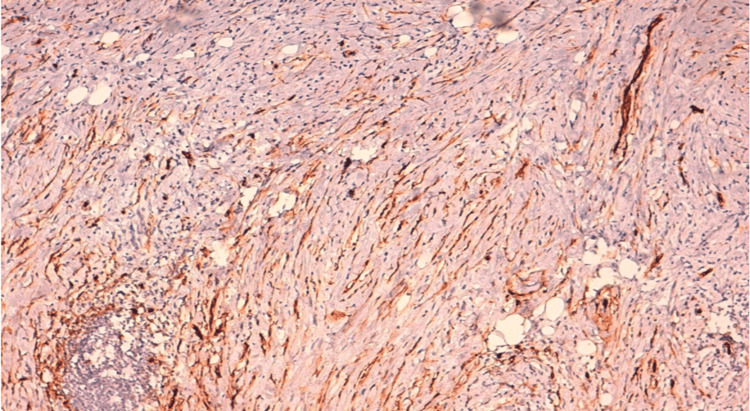
Focal expression of CD34 by tumor cells. GAB X10

**Figure 3:**
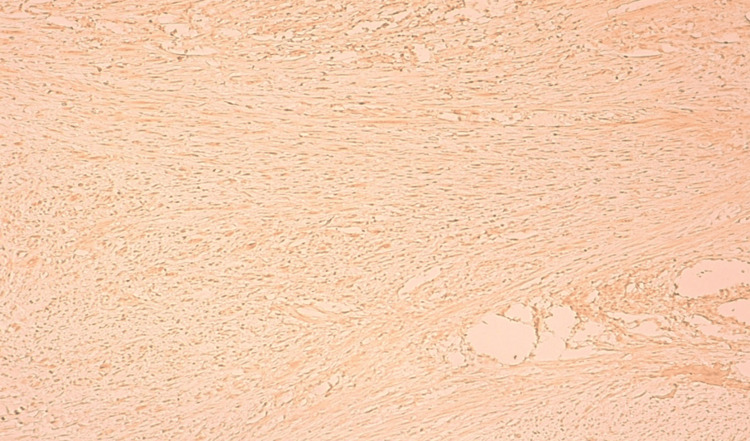
Focal expression of desmin by tumor cells. GAB X10

The patient underwent a wide resection of the mass with microscopic positive margins. According to the primary anatomopathological study, a proliferation of fasciculated architecture, consisting of cells exhibiting mild cytonuclear atypia with elongated, wavy nuclei and tapered ends, sparse, eosinophilic cytoplasm, and frank focal atypia with enlarged, irregular, and hyperchromatic nuclei, was found in the deep and superficial dermis. The estimated quantity of mitoses is seven mitoses/10 fields, as observed at high magnification. The stroma in certain areas was found to be fibromyxoid and not excessively inflammatory. According to immunohistochemistry, the tumor cells exhibited positive staining with PS100 and SOX10, as well as focal expression of CD34 and desmin (Figures 2, 3 ).

**Figure 4:**
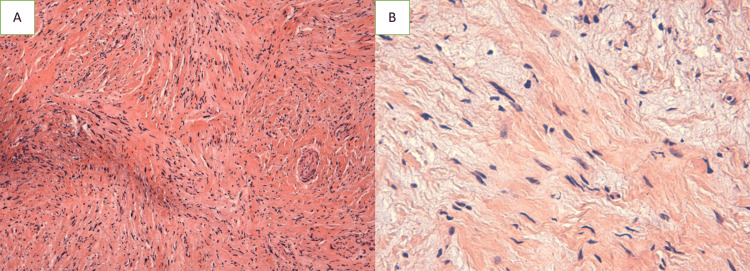
Spindle cell proliferation with elongated, hyperchromatic nuclei, separated by thickened bands of collagen, with focal desmoplastic reaction. H&E x10 (A) and H&E x20 (B)

The diagnosis of a peripheral nerve sheath tumor was maintained. Given the rarity of this entity and its association with chemoresistance, a review of the case was conducted in the multidisciplinary consultation meeting. The histology and immunohistochemistry results were reexamined to provide a clearer understanding of the role of adjuvant chemotherapy. This revision revealed the growth of spindle-cell tumors that originated in the reticular dermis and invaded the hypodermis. They displayed mild to moderate cytonuclear atypia, elongated nuclei, and scant eosinophilic cytoplasm (Figure 4 ).

**Figure 5:**
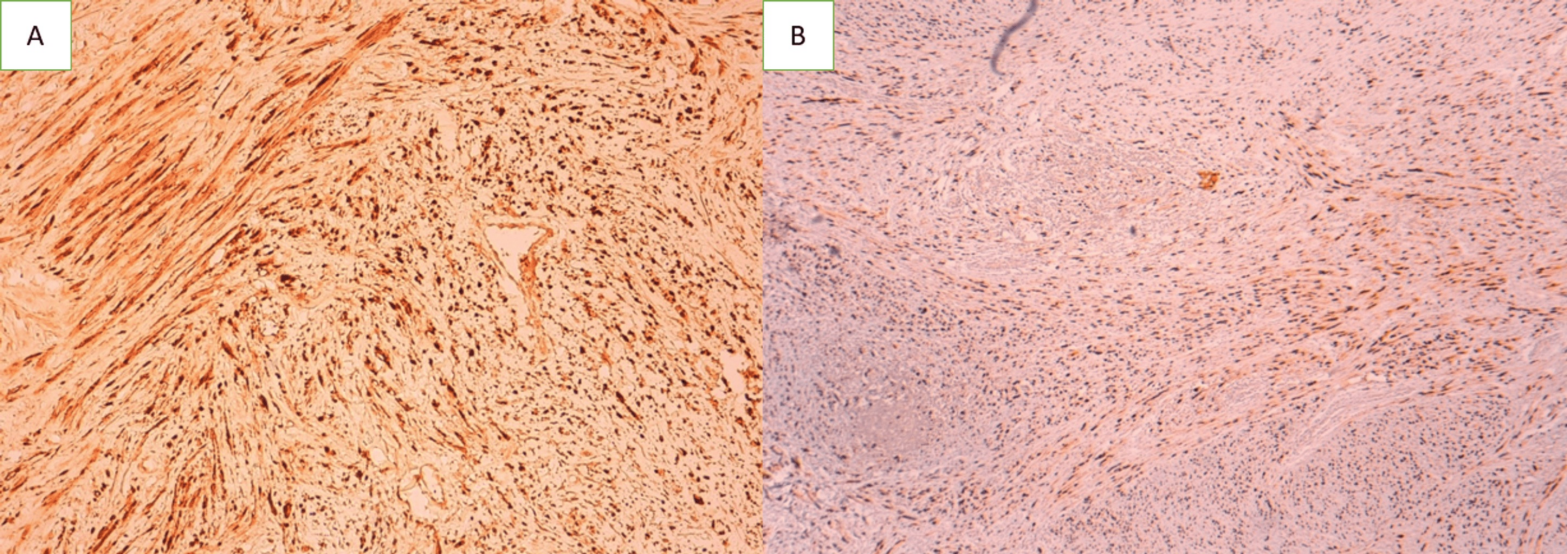
Strong and diffuse expression of S100 (GAB X10) (A) and SOX 10 (GAB X4) (B) by tumor cells

The PS100 and SOX10 tests showed widespread, strong positive staining (Figure 5), and the Ki67 estimate was 40%. As a result, the histological diagnosis of a desmoplastic malignant melanoma was made.

The patient received adjuvant radiotherapy and is currently being monitored with good locoregional development.

## Discussion

DM is a rare histological subtype (less than 4% of all cutaneous melanomas) [5]. Men are more often affected than women, with a sex ratio of 2:1. The average age ranges from 55.6 to 71.3 [13,14]. DM was described for the first time by Conley et al. as a particular and aggressive form of melanoma in 1971 [6]. It begins as a superficial melanotic lesion and develops into an aggressive tumor that invades surrounding tissue with metastatic potential.

Clinically, DM manifests as a painless, indurated plaque that progresses slowly, however, some lesions may begin as a tiny papule or macule. They are frequently unpigmented unless there is lentigo maligna in the surrounding epidermis, which occurs in around 50% of cases [15]. Unfortunately, the clinical diagnosis of DM is frequently overlooked, leading to diagnostic delays. It is mistaken for other cancerous lesions like solar keratosis, squamous cell carcinoma, amelanotic melanoma, and basal cell carcinoma, as well as benign lesions like scars and nevi [16].

Histologically, the lesions are typically poorly confined, affecting the entire dermis and frequently extending to the subcutaneous tissue on severely sun-damaged skin. They are mainly made up of asymmetric, deeply invading spindle cells. In most cases, these lesions are microscopically amelanotic, which is likely the primary reason why histological diagnosis of DM is not successful in most patients [13,16]. The desmoplastic component refers to the presence of collagen bundles surrounding spindle cells in a fibrotic or fibromyxoid stroma, giving the melanocytes a paucicellular appearance [17]. Usually, the stroma is associated with little lymphocyte islands and occasionally a few plasma cells within and/or at the edge of the tumor, which might be a crucial diagnostic lead [18]. Neurotropism is an additional intriguing histological characteristic of DMs. This term is used to describe melanocytes found around or within small peripheral and/or deep nerve bundles in proximity to the tumor mass [7]. It is crucial to comprehend the clinico-histological characteristics of DMs in order to address the diagnostic challenges that may cause delays or even incorrect diagnoses.

Five cases in the collection by Lorcy and colleagues had histological diagnoses that were deemed challenging, which prompted discussions about sarcomas, histiocytofibromas, a Spitz nevus, and peripheral nerve sheath tumor [14].

Generally speaking, though, DM poses more of a diagnostic than a differential diagnosis problem. In other words, the diagnostic error typically results from failing to recognize the tumor as a likely candidate for diagnosis rather than actively rejecting it in favor of a different theory or alternative [19]. For this specific reason, immunohistochemistry is commonly indicated. In particular, S100 is diffusely positive; however, staining may be more restricted in certain DM patients [18]. Other more commonly used markers of melanocytic differentiation such as HMB45 and Melan A are generally negative in the paucicellular components of spindle cells in DM [20]. In recent years, promising markers have been created in this regard, including the SOX-10 marker and the p75 nerve growth factor receptor (NGFR). The latter has shown a higher rate of positivity in desmoplastic and spindle cell melanomas [21]. Furthermore, a number of studies have demonstrated improved specificity for differential diagnosis, specifically in separating DM from scar lesions, and higher sensitivity for SOX 10 in comparison to S100 in the diagnosis of DM [22]. Therefore, the application of these markers may enhance diagnostic sensitivity while assisting pathologists in guaranteeing a more precise and dependable diagnosis which continues to be the true difficulty for them [21].

The molecular biology or genetic profile of DM is distinguished by a high rate of mutation, which is significantly different from that of conventional melanoma, with a reduced number of genes involved in melanin synthesis, explaining the amelanotic character of the lesions, and an increase in the expression of neurotrophic factor genes and genes linked to matrix production, which is also consistent with the phenotypes attributed to DM [23]. BRAF, NRAS, and C-KIT mutations are, nevertheless, often less common in DM than in other forms of melanoma [24,25].

First-line treatment of any primary cutaneous melanoma, as well as DM, is based on wide surgical resection, offering a chance of cure and regional disease control [26,27]. The initial tumor's thickness determines the excision margin. Therefore, 1-2 cm is the recommended margin for a lesion that is 1-2 mm thick; for melanomas that are over 2 mm, a 2 cm margin is suggested, and for those that are less than or equal to 1 mm, a 1 cm margin is sufficient [28]. Following these recommendations is crucial, since the extent of surgical resection in MD, regardless of tumor thickness, remains a significant predictor of survival, as demonstrated by a large series study of Surveillance, Epidemiology, and End Results (SEER) cases, with minimum acceptable resection margins of 1 cm [29].

The topic of sentinel lymph node biopsy is complicated, and opinions on its usefulness are still divided. To choose patients who are suitable for this kind of surgery, a multidisciplinary discussion is necessary [19].

Adjuvant radiation has become an intriguing part of the therapeutic approach to DM in order to reduce the risk of local recurrence at surgical excision sites. Retrospective studies have shown that adjuvant radiation significantly lowers the rate of local recurrence in patients with operated DM who are not able to achieve R0 resection [30].

On the other hand, a positive response to immunotherapy is linked to the high mutational burden found in this tumor [31]. In fact, in the era of precision medicine, the emergence of immune checkpoint inhibitors as a promising approach has opened the way to a wave of therapeutic options in several malignant cancers [32].

The benefit of immunotherapy in DM was assessed in a retrospective study involving 60 patients, with an objective response rate of 70% [31]. The phase 2 SWOG 1512 study is the first randomized trial dedicated to investigating the response to immunotherapy, with two cohorts: the first included patients with resectable DM, while the second consisted of unresectable DM. Recent data for the first cohort show that this entity responds well to pembrolizumab monotherapy, with an overall response rate of 89% [33], while neoadjuvant pembrolizumab achieved substantial pathological complete response (pCR) rates, once again confirming the sensitivity of these tumors to immunotherapy [34].

## Conclusions

DM is a rare and distinct subtype of melanoma that commonly affects sun-damaged skin. It poses a significant challenge to clinicians and pathologists due to its indolent clinical presentation with no specific appearance, which can lead to misinterpretation under the microscope. In the era of precision medicine, it is imperative to apply further investigation to overcome the diagnostic pitfalls and aid practitioners in adopting optimal management strategies. Elucidating the biomarkers and molecular genetic profiling of DM may pave the way for new targeted therapies, as promising results have been demonstrated with targeted immunotherapies.
